# An observational study of the quality of care for chronic kidney disease: a Buffalo and Albany, New York metropolitan area study

**DOI:** 10.1186/s12882-015-0194-2

**Published:** 2015-12-03

**Authors:** Pradeep Arora, Peter L. Elkin, Joseph Eberle, J. James Bono, Laura Argauer, Brian M. Murray, Raghu Ram, Rocco C. Venuto

**Affiliations:** State University of New York at Buffalo, School of Medicine and Biosciences, Buffalo, NY 14215 USA; Computer Task Group, Inc, 800 Delaware Avenue, Buffalo, NY 14209 USA; HealthNow New York, 257 West Genesee Street, Buffalo, NY 14202 USA; Nephrology Department, 462 Grider Street, Buffalo, NY 14215 USA

**Keywords:** Chronic kidney disease, Quality of care assessment, Claims data mining

## Abstract

**Background:**

The database of a major regional health insurer was employed to identify the number and frequency of covered patients with chronic kidney disease (CKD). We then examined the characteristics of their care as defined, in part, by the frequency of physician visits and specialty referral, the characteristics of laboratory testing and total costs as indices of the quality of care of the subject population.

**Methods:**

This retrospective, cross-sectional study analyzed insurance claims, laboratory results and medication prescription data. Patients with two estimated glomerular filtration rate readings below 60 ml/min/1.73 m^2^ (*n* = 20,388) were identified and classified by CKD stage.

**Results:**

The prevalence of CKD stages 3a and above was 12 %. Vascular comorbidities were common with prevalence increasing steadily from stage 3a through stage 5. Only 55.6 % of stage 4 CKD patients had claims for nephrology visits within one year of their index date. Fifty-nine percent of patients had claims for renin-angiotensin system (RAS) blockers. Twenty-five percent of patients in stage 3a CKD filled a prescription for non-steroidal anti-inflammatory drugs. Fifty-two percent of patients who developed end-stage renal disease received their first dialysis treatment as inpatients.

**Conclusions:**

The pattern of medical practice observed highlights apparent deficiencies in the care of CKD patients including inappropriate medication use, delayed nephrology referral, and a lack of preparation for dialysis. This study shows the potential value of using large patient databases available through insurers to assess and likely improve regional CKD care.

## Background

Chronic kidney disease (CKD) is a major public health problem that is associated with increased morbidity, mortality, and healthcare costs for both individual patients and health care systems [1–3]. The health burden caused by CKD is likely to grow sharply over the next several years due to an increasing elderly population and the escalating prevalence of comorbid conditions such as type II diabetes. Indeed, the United States Renal Data System (USRDS) reported a 104 % increase in the prevalence of end-stage renal disease (ESRD) between 1990 and 2002 [4, 5]. Therefore, it is crucial to monitor the population at risk.

Mining high-dimensional insurance claim data can yield population-level insights on epidemiology, outcomes and economics of a particular disease entity [6, 7]. Quality data of the sort provided during the processing of health insurance claims could offer a unique window into CKD prevalence, treatment modalities, and outcomes. Diagnostic testing rates, prevalence of comorbidities, and management strategies relating to CKD have yet to be described for the Albany and Buffalo metropolitan areas of New York. Consequently, we analyzed the insurance claims for more than one million individuals residing primarily in these areas to assess the regional recognition and care of CKD patients.

The agenda of many Federal funding agencies such as the Patient Centered Outcomes Research Institute (PCORI) is to determine best practice for using observational data in clinical research. This study provides important quality data for the care of chronic kidney disease. Studies applied to observational data have the disadvantages of lacking both randomization and structure in the data collection process. Nonetheless, they are truly in vivo studies where the practice settings are representative of reality. They also have the advantage that the number of patients included in the trial is often larger: this affords the observational study increased power to identify important clinical outcomes.

## Methods

### Study design and sample

The study population for this report was derived from a large database of health insurance claims records from a major insurer serving the greater Metropolitan Buffalo and Albany regions of New York State. These data were gathered between January 2007 and August 2013. Data collection was initiated by the University at Buffalo School of Medicine and Biomedical Sciences. Use of the data for analysis and interpretation was approved by the institutional review board of the School of Medicine of the State University of New York at Buffalo. All of the authors of this manuscript were HIPPA certified. Since all data was de-identified individual informed consent was not obtained. Original data for this manuscript resides in the Institute for Health Information of the School of Medicine and Biomedical Sciences. Access to this date is restricted to only the investigators.

**Fig. 1:**
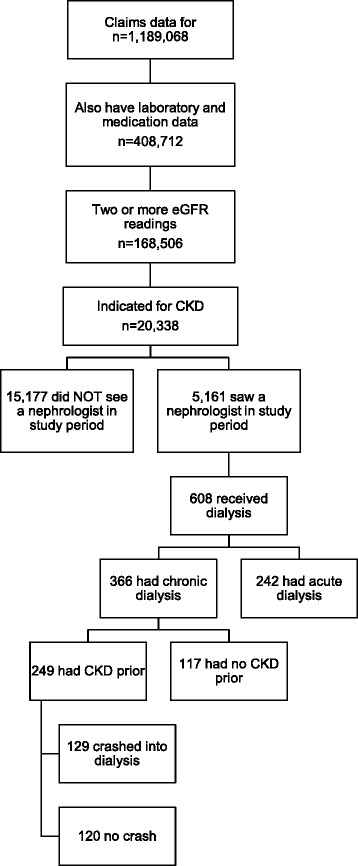
Flowchart of the study population

The database did not contain information for subscribers for whom the insurer had not received claims; therefore, every patient (*n* = 1,189,068) had at least one insurance claim. Of these individuals, only 168,506 patients had all of the data necessary to identify medications and laboratory results required for our analysis of that group. Patients with eGFR readings below 60 ml/min/1.73 m^2^ on two occasions at least 90 days apart (*n* = 20,388) were classified by CKD stage (Fig. 1).

Patients were assigned index dates set at the date of their second abnormal eGFR reading. Each patient was also assigned a unique identification number that allowed patient data to be aggregated across all claim types (pharmacy, professional, inpatient, outpatient, and laboratory), all of which were stored in separate database tables.

### Baseline data identification and collection

We used the standard coding tools to extract information from the data set, including Current Procedures and Terminology (CPT); International Classification of Diseases, Ninth Revision, Clinical Modification (ICD-9CM); Logical Observation Identifiers Names and Codes (LOINC); National Provider Identification (NPI); and National Drug Codes (NDC).

We utilized Computer Task Group’s HIE Lite platform to aggregate data from several disparate data sources including medical claims, laboratory results, pharmaceutical claims, and patient demographics. After normalization of the dataset we were able to develop new models to examine the temporal aspects of disease progression and identify the critical gaps in care and most significant medical events.

### Prevalence rates for select comorbid conditions

ICD-9CM diagnosis codes were classified according to the Agency for Healthcare Research and Quality’s Clinical Classification Software categories for coronary artery disease (CAD), congestive heart failure (CHF), peripheral vascular disease, cerebrovascular accidents (CVA), chronic obstructive pulmonary disease (COPD), malignancy, hypertension, diabetes mellitus, and mental illness. Comorbid conditions were considered “present” in a patient if there was at least one qualifying diagnosis code in their record.

### CKD prevalence

CKD was defined according to National Kidney Foundation–Kidney Disease Outcomes Quality Initiative (NKF–KDOQI) guidelines by the recording of two determinations separated by at least three months of eGFRs of less than 60 ml/min/1.73 m^2^ using the Modification of Diet in Renal Disease equation. The prevalence of CKD was calculated by dividing the number of patients meeting the definition of CKD (*n* = 20,388) by the number of patients who had two calculated eGFR and claims records for medications and laboratory data (*n* = 168,506).

### Laboratory data

Laboratory tests for iron profile (iron, total iron binding capacity, and ferritin), albumin, albumin/creatinine ratio (ACR), hemoglobin A1c (glycosylated hemoglobin), 25-hydroxy Vitamin D_3_, phosphorus, calcium, intact parathyroid hormone (PTH), and lipid level results were identified using LOINC and tracked throughout the observed period.

### Prescription information

NDC numbers were used to identify medications that corresponded with classes of interest. Specifically, renin-angiotensin system (RAS) blockers, phosphate binders, active Vitamin D analogs, erythropoesis-stimulating agents (ESAs), anti-hyperlipidemic, and antihypertensive agents were recorded at baseline and at the end of the study period.

### Renal “crash”

We defined renal crash patients were those who met all of the following criteria: 1. developed ESRD during the observed period; 2. experienced their initial dialysis in an inpatient setting; 3. had at least two eGFR values indicative of CKD (less than 60 ml/min/1.73 m^2^) before their first dialysis; and 4 had dialysis claims persisting for 90 days after the initial dialysis billing (Fig. 1).

### Statistical analysis

Data analysis consisted of using simple frequencies with percentages to determine CKD prevalence, comorbid condition prevalence, medication use, laboratory testing, and nephrologist visits within the qualifying population. Descriptive statistics such as mean, median, and standard deviation were used for continuous variables. Pearson’s Chi-square test was applied to find associations between two categorical variables. Student’s *t*-test and Kruskal-Wallis non-parametric ANOVA were used to determine significant differences between average values across groups. All calculations were performed using v3.1.0 of the R language and environment for statistical computing.

## Results

### Population characteristics

CKD prevalence in the study population with the requisite available lab data was 12 % (20,338 patients out of the 168,506 patients with two or more eGFR readings). Sixty- one per cent of the CKD patients were female. Eighty-two percent of patients were over 60 years of age and the median age was 72 years.

### Comorbidities

The prevalence of comorbidities by CKD stage was evaluated. Hypertension was present in 89.0 % of patients and 42.3 % had diabetes. Cardiovascular diseases such as CAD, CVA, and CHF were common and their prevalence increased from 20 % in CKD stage 3a to 35 % in CKD stage 5. Patients with CKD also exhibited a relatively high rate (29.5 %) of non-dermatologic cancer diagnoses. The other comorbidities commonly reported in CKD cohort were depression (17 %), anxiety (15 %), other mental illnesses (28 %), and substance abuse (3 %). In contrast to cardiovascular complications, the rates of behavioral health conditions and cancer did not rise substantively as CKD progressed.

### Provider encounters

Table 1 presents the annualized percentage of patients who made at least one visit to a primary care physician (PCP) or a nephrologist during the 12 months after their first appearance in the data set.

**Table 1:** Annualized percentage of visits to PCP and nephrologist

CKD stage	No. of patients	Patients who saw either a PCP or nephrologist	Patients who saw a PCP (%)	Patients saw a nephrologist (%)
3a	13,648	9598	9443 (69.2)	628 (4.6)
3b	5178	3884	3705 (71.6)	865 (16.7)
4	1292	1069	966 (74.8)	575 (44.5)
5	220	204	183 (83.2)	15 (70.9)
All	20,338	14,755	14,297 (70.3)	2224 (10.9)

*Abbreviations*: *CKD* chronic kidney disease, *PCP* primary care physician

**Fig. 2:**
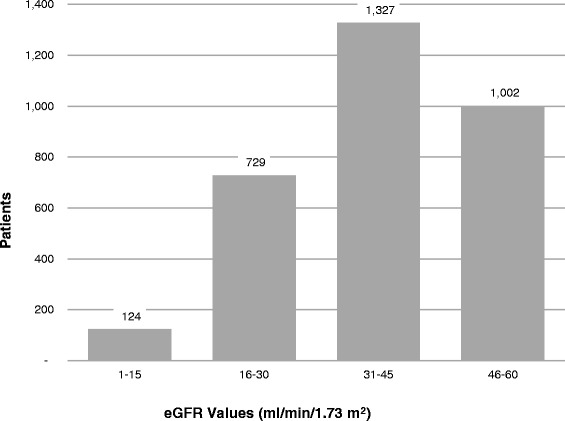
Distribution of eGFR values at first nephrology visit eGFR. Values (ml/min/1.73 m^2^)

Nephrologists saw 14.5 % of patients within one year of their index date: 6.6 % in stage 3a, 21.7 % in stage 3b, 55.6 % stage 4, and 87.7 % in stage 5. The mean eGFR at the time of first nephrology visit was 39.2 (+/− 15.54) ml/min/1.73 m^2^. The distribution of eGFR values at the time of first nephrology claim is presented in Fig. 2.

### Screening and prevalence for associated conditions

Table 2 depicts the frequency with which testing recommended for patients with CKD was performed.

**Table 2:** Frequency of laboratory tests

CKD related lab tests	Percent of CKD patients who had lab test and did or did not see a nephrologist, by stage									
	Stage 3a		Stage 3b		Stage 4		Stage 5		All stages	
	(*n* = 13,648)		(*n* = 5178)		(*n* = 1292)		(*n* = 220)		(*n* = 20,338)	
	No	Yes	No	Yes	No	Yes	No	Yes	No	Yes
Hemoglobin	84.9 %	92.8 %	85.3 %	93.9 %	83.6 %	95.3 %	92.9 %	89.3 %	85.0 %	93.5 %
Iron	15.7 %	34.1 %	19.6 %	42.6 %	20.4 %	55.1 %	33.7 %	41.3 %	16.6 %	41.4 %
Total iron binding capacity	12.8 %	31.3 %	4.4 %	39.9 %	0.6 %	53.5 %	<0.01 %	39.8 %	13.6 %	38.8 %
Ferritin	13.8 %	30.7 %	17.7 %	38.2 %	15.6 %	51.9 %	21.4 %	38.8 %	14.7 %	37.6 %
Calcium	99.6 %	99.6 %	99.5 %	99.8 %	98.9 %	99.8 %	100.0 %	100.0 %	99.6 %	99.7 %
Phosphate	6.0 %	52.7 %	7.6 %	64.0 %	10.8 %	73.9 %	24.1 %	60.8 %	6.5 %	61.5 %
Vitamin D	47.2 %	54.4 %	42.6 %	56.3 %	36.8 %	54.9 %	21.4 %	35.4 %	45.9 %	54.5 %
PTH	6.5 %	35.8 %	8.3 %	51.9 %	10.6 %	64.2 %	7.1 %	49.0 %	7.0 %	47.5 %
Albumin	95.2 %	97.4 %	93.8 %	97.3 %	93.4 %	96.7 %	100.0 %	91.7 %	94.9 %	97.0 %
ACR	28.9 %	49.0 %	8.2 %	49.5 %	0.9 %	44.5 %	<0.01 %	23.8 %	29.0 %	47.4 %
A1c	49.3 %	62.3 %	49.1 %	61.6 %	47.4 %	58.1 %	50.0 %	45.1 %	49.2 %	60.6 %

*Abbreviation*: *A1c* glycosylated hemoglobin, *ACR* albumin-creatinine ration, *CKD* chronic kidney disease, *PTH* parathyroid hormone

Both screening for and prevalence of conditions associated with CKD varied widely depending on nephrology referral and CKD stage. Patients were more likely to have had tests for metabolic bone disease and anemia if they were seen by a nephrologist. Goal achievement for selected labs prescribed by NKF–KDOQI guidelines varied with CKD stage.

### Prescribing patterns

The prescription claims for medications recommended to be employed or avoided in patients with CKD are depicted in Table 3.

**Table 3:** Prescription drug usage in CKD patients

CKD-related medication	CKD patients who took medication and did or did not see a nephrologsit (%), by stage							
	Stage3a		Stage 3b		Stage 4		Stage 5	
	No	Yes	No	Yes	No	Yes	No	Yes
	(*n* = 11,583)	(*n* = 2065)	(*n* = 3202)	(*n* = 1976)	(*n* = 378)	(*n* = 914)	(*n* = 14)	(*n* = 206)
RAS blocker	6478 (55.9)	1390 (67.3)	1975 (61.7)	1312 (66.4)	204 (54.)	516 (56.5)	4 (28.6)	89 (43.2)
Aldosterone antagonist	440 (3.8)	252 (12.2)	210 (6.6)	276 (14.0)	33 (8.7)	111 (12.1)	1 (7.1)	5 (2.4)
Phosphate binder	4 (<0.1)	26 (1.3)	0 (0)	71 (3.6)	5 (1.3)	157 (17.2)	2 (14.3)	120 (58.3)
Vitamin D	588 (5.1)	256 (12.4)	113 (3.5)	291 (14.7)	15 (4.0)	272 (29.8)	1 (7.1)	78 (37.9)
PPI	3592 (31.0)	810 (39.2)	1087 (33.9)	812 (41.1)	140 (37.0)	394 (43.1)	3 (35.7)	85 (41.3)
ESA	10 (0.1)	39 (1.9)	15 (0.5)	88 (4.5)	6 (1.6)	123 (13.5)	0 (0)	38 (18.4)
Loop diuretics	2193 (18.9)	809 (39.2)	1029 (32.1)	958 (48.5)	147 (38.9)	572 (62.6)	4 (28.6)	99 (48.1)
Statin	6056 (52.3)	1249 (60.5)	1721 (53.7)	1197 (60.6)	179 (47.4)	539(59.0)	5 (35.7)	110 (5.4)
NSAID	2939 (25.4)	492 (23.8)	662 (20.7)	392 (19.8)	45 (11.9)	125 (13.7)	2 (14.3)	11 (5.3)

*Abbreviations*: *CKD* chronic kidney disease, *ESA* erythropoeisis-stimulating agent, *NSAID* non-steroidal anti-inflammatory drug, *PPI* proton pump inhibitor, *RAS* renin-angiotensin system

Only 58.8 % of patients had claims for RAS blockers. RAS blocker use was greater in early stages of disease and in those seeing a nephrologist. The use of ESAs increased from 0.4 to 17.3 % from stage 3a to stage 5. Use of phosphate binders, active Vitamin D analogs, and ESAs increased with severity of CKD and with nephrology visits. Twenty-five percent of patients in stage 3a CKD filled a prescription for non-steroidal anti-inflammatory drugs (NSAIDs). Even some patients in stage 5 were on prescribed NSAIDs (5.9 %). Filled prescription rates for NSAIDs were significantly lower in the collective CKD population seen by nephrologists (19 vs. 24 %, *P* < 0.005). There was, nonetheless, a substantial number of patients on prescribed NSAIDs even among those seen by nephrologists.

### Preparation for dialysis

Overall, 366 patients in the entire study population (*n* = 168,506) started chronic dialysis for ESRD; of these patients, 249 had documented CKD prior to the initiation of dialysis. Only 26 % of these 249 patients had an arteriovenous fistula when they first received dialysis. The initial dialysis occurred in an inpatient setting for 51.8 % of these patients. Sixty-three percent of patients who “crashed” had claims for initial nephrology visits more than one year prior to starting dialysis, 13.9 % had claims between 90 days and one year prior to dialysis, 10 % had claims less than 90 days prior, and 13.1 % had no nephrologist claims before their crash [Table 4].

**Table 4:** Timeframe of Nephrologist claims prior to crash

	CKD patients who crashed (%)	CKD patients who did not crashed (%)
	(*n* = 129)	(*n* = 120)
Initial claim:		
More than i year prior to crash	81 (62.7)	79 (65.8)
90 days to 1 year prior to crash	18 (13.9)	27 (22.5)
Fewer than 90 days prior to crash	13 (10)	8 (6.7)
After first dialysis	17 (13.1)	6 (5)
Last claim:		
More than i year prior to crash	6 (4.6)	4 (3.3)
90 days to 1 year prior to crash	14 (10.8)	4 (3.3)
Fewer than 90 days prior to crash	92 (71.3)	106 (88.3)
After first dialysis	17 (13.1)	6 (5)

*Abbreviations*: *CKD* chronic kidney disease

In the subgroup of patients who were found to have a renal crash, those who saw a nephrologist in the last year were compared with those who did not see a nephrologist. Crashes were less frequent when patients had an encounter with a nephrologist in the last year (48 vs. 68 %, *P* < 0.005).

## Discussion

In a selected group of 168,506 insured individuals who had at least two qualifying eGFR readings separated by two months and claims records for medications and laboratory studies, the prevalence of CKD stage 3 and above was 12 % (20,388). The pattern of medical practice observed in this study suggests that substantial opportunities exist to improve care for this population. Several findings are noteworthy: first, the proportion of patients with CKD who had claims for care by a nephrologist (24 %) was smaller than expected, but increased with the severity of CKD. The annualized percentage of patients who visited nephrologists increased as kidney function worsened, yet only 55.6 % of patients with stage 4 CKD had a claim from a nephrologist. Second, the percentage of patients receiving laboratory tests recommended by the NKF–KDOQI standard was low, even in those patients seen by a nephrologist. Third, the level of prescription of RAS blockers was relatively low, though higher when patient had a nephrology claim (64.1 vs. 57.1 %, *P* < 0.005) and declined with advancing CKD (stage 3, 60 %; stage 4, 55.7 %; stage 5, 42.3 %). Fourth, a substantial number of patients in the more advanced stages of CKD (stages 4 and 5) were prescribed potentially nephrotoxic NSAID medications. Finally, among patients who developed ESRD, 51.8 % had their first instance of dialysis as inpatients and only 26 % had evidence that an arteriovenous fistula had been placed.

CKD patients had a high frequency of comorbidities similar to what has been reported [8]. The higher rates of various vascular comorbidities in CKD patients could be related to a higher prevalence of diabetes, hypertension, elevated triglycerides and low high-density lipoprotein levels, increased oxidative stress, inflammation, physical inactivity, anemia, vascular calcification, and left ventricular hypertrophy. Another important finding in the study is the higher than expected prevalence of cancer in CKD patients. In a recent study Wong et al. identified moderate CKD (stage 3) was identified as an independent risk factor for the development of cancer among older men [9]. A causal relationship between cancer and CKD has not been established. It is possible, however, that CKD patients may have a higher cancer rate related to decreased immune surveillance; alternatively, patients with cancer may be more prone to CKD due to unrecovered acute kidney injury secondary to cancer itself and/or nephrotoxicity from chemotherapy or other cancer treatments [9–12].

Improving management of CKD during its early stages can reduce the growing burden of ESRD. Although there are guidelines developed by medical experts that define best practices for early-stage CKD management, their implementation may not be optimal and result in poor outcomes [4, 13–15]. Results from the NKF’s Kidney Early Evaluation Program study revealed poor control of cardiovascular risk factors among CKD patients [8]. Appropriately managing anemia, hypertension, and dyslipidemia could also help prevent cardiovascular mortality, which is a leading cause of death among CKD patients. In this population, iron saturation tests were performed in only 23 % of patients with CKD and PTH levels were assessed in only 49 % of patients in CKD stage 4. Although these tests were performed more frequently when nephrologists were involved in patient care (Table 3), testing rates still fell short of guideline recommendations.

Interventions to slow progression are critical to the effective treatment of CKD and prevention of ESRD. RAS blockers have been shown to retard the progression of kidney disease [16–19]. The American College of Physician (ACP) guidelines for CKD care recommend prescribing RAS blockers to every CKD patient unless contraindicated [20]. Patients in this study who saw a nephrologist were more likely to be prescribed RAS blockers (4 vs. 57.1 %, *P* < 0.005). In a study of patients in the greater metropolitan Boston, Massachusetts, area more than 10 years ago, Kausz, et. al. reported that RAS blockers were used in 49 % of patients when seen by a nephrologist [21]. We found RAS blocker use was far from the now even higher rate recommended for those without contraindications but greater than that noted in the 2000 Boston study [13–15]. There still may be room for improvement: in Singapore for example, a more controlled medical system, Ang et. al^.^ found in 2011 that 84 % of CKD patients were prescribed RAS blockers [22].

It is clinically sound practice for CKD patients to avoid nephrotoxic medications such as NSAIDs despite conflicting study results linking these medications and kidney disease progression [23–25]. In a study that employed NHANES data, Plantinga, et. al. examined NSAID use among CKD patients [23]. Using a questionnaire method, they found that 5 % of patients with moderate to severe CKD reported NSAID use. In this population NSAID use was much higher from 25 % in stage 3a down to 5.9 % in stage 5. When combined with RAS blockers, which are commonly prescribed to elderly patients with hypertension, NSAIDs may increase the risk of superimposed acute kidney injury. This point alone makes the level of continued NSAID prescription revealed by this study a topic of concern. Clinicians may have felt that the presence of comorbid conditions warranted the use of NSAIDs to achieve better quality of life despite the inherent risks. Although patients under nephrology care filled fewer claims for NSAIDs than those seen by PCPs alone, any NSAID prescription for a patient with advanced CKD who is receiving nephrology care is worrisome. It is not always possible from our database to identify a prescribing practitioner’s specialty. Furthermore, because claims data do not generally include over-the-counter medications, our results likely underestimate NSAID use in the study population.

Despite improvements in dialysis care and increased spending on healthcare, the mortality rate among ESRD patients remains high. Several factors, including delayed referral to nephrologists, appear to influence mortality among dialysis patients [26–31]. Late referrals reduce the likelihood of treatment for anemia, dyslipidemia, renal bone disease and the timely placement of appropriate vascular access, preemptive kidney transplant, or informed selection of dialysis modality. Indeed, earlier involvement of the nephrologist was associated in one study with lower subsequent patient mortality [28].

The NKF recommends that patients consult a nephrologist when their eGFR dips below 30 ml/min/1.73 m^2^. Late nephrology referral is a widespread problem, as described by Sprangers, et. al. who claim of up to 84 % of patients are referred late [30]. The analysis of our data set shows that 23 % of patients had a late nephrology referral. Referral to nephrology did not necessarily obviate “crashing” into dialysis, since 50 % of patients who were seen by nephrologists three months prior to dialysis received inpatient dialysis, compared to 75 % who were not seen by a nephrologist in three months. Indeed, 71 % had seen their nephrologist within 90 days of the crash. We also found that 80 % of patients who experienced a renal “crash” required a central venous catheter as vascular access. Nonetheless, crashes were less frequent when patients saw a nephrologist (48 vs. 68 %, *P* < 0.005). The reasons for urgent inpatient dialysis and delayed or missing claims for nephrology, however, could not be established in this study.

There are several limitations to this study, some of which are inherent to using claims data in population studies. We were unable to obtain data on patients’ race from the provider. As per NKF–KDOQI guidelines, we identified CKD using two values of serum creatinine, taken three months apart. The definition of CKD for this study was based on eGFR readings below 60 ml/min/1.73 m^2^. As a result, it excluded patients with CKD stages 1 and 2, which, according to NHANES data, constituted about one-third of all patients with CKD [32]. The requisite laboratory data to establish the presence of CKD was available on only about 15 % of the nearly 1.2 million insured individuals. Within that group the incidence of CKD stages 3 or higher was 12 %. This is higher than the 8 % reported by NHANES, but the difference could be explained by the fact that patients at risk for CKD are more likely to have the relevant laboratory tests [2]. Indeed, the patients we studied were defined based on criteria that included sufficient laboratory testing to confirm CKD and could result in an apparently higher prevalence.

Another limitation is that claims data, by its nature, does not include information on uninsured patients and our analyses may not be applicable to their care, which may be less robust. The intention of this study, however, was not to report on a representative cohort of the population but rather to characterize the care received by patients with clearly established CKD. One of our study’s strengths is that we included all the claims from a major regional insurance provider, as well as available laboratory data, and did not limit to a specific group alone. In the future, electronic health record data could be added to the claims data to improve our ability to surveil patient conditions and outcomes [32, 33]. Studies applied to observational data typically lack both randomization and structure in the data collection process. They are, however, truly in vivo studies where the practice settings are representative of reality and often include a larger patient base.

## Conclusions

In conclusion, we were able to document striking deviations from the quality of care guidelines in terms of nephrology referral timing, recommended medication use, and the frequency with which chronic dialysis was initiated in under- or unprepared patients. These findings demonstrate that regional insurance claim data can provide information useful to assess the prevalence and treatment of CKD. The analyses of treatment patterns presented in this study indicate times and settings where better education, information sharing, and adherence to guidelines can make real improvements in CKD patient care, particularly where nephrotoxic medications, specialist referral, and preparation for renal replacement therapy are concerned. Future initiatives to consolidate and enrich such efforts should become the hallmarks of CKD care in Buffalo, Albany, and beyond.

## Abbreviations

ACR: albumin/creatinine ratio
CAD: coronary artery disease
CHF: congestive heart failure
CKD: chronic kidney disease
COPD: chronic obstructive pulmonary disease
CPT: Current Procedures and Terminology
CVA: cerebrovascular accidents
eGFR: estimated glomerular filtration rate
ESAs: erythropoiesis-simulating agents
ESRD: end-stage renal disease
glycosylated hemoglobin: hemoglobin A1c
ICD-9CM: International Classification of Diseases, Ninth Revision, Clinical Modification
LOINC: Logical Observation Identifiers Names and Codes
MDRD: Modifications of Diet in Renal Disease
NDC: National Drug Codes
NKF–KDOQI: National Kidney Foundation–Kidney Disease Outcomes Quality Initiative
NPI: National Provider Identification
NSAIDs: non-steroidal anti-inflammatory drugs
PCORI: Patient Centered Outcomes Research Institute
PCP: primary care physician
PTH: intact parathyroid hormone
RAS: renin-angiotensin system
USRDS: United States Renal Data System
